# OA30 Potential Erythromelalgia or Fabry’s in Young Lady with Hypermobility

**DOI:** 10.1093/rap/rkae117.030

**Published:** 2024-11-05

**Authors:** Susan Maillard, Katy Edebol, William Trapp

**Affiliations:** Great Ormond Street Hospital NHS Foundation Trust, London, United Kingdom; Great Ormond Street Hospital NHS Foundation Trust, London, United Kingdom; Great Ormond Street Hospital NHS Foundation Trust, London, United Kingdom

## Abstract

**Introduction:**

This case is an example of a complex patient who presents with pain and other symptoms and after assessment and investigations has a combination of complex pathology as this is suspected to be Erythromelalgia or Fabry’s disease as well as hypermobility and have lost function and independence and when they first attended clinic were in a wheelchair and using crutches. The challenges of the case are working with the family and young person to enable them to manage the pain effectively whilst regaining independent mobility and function and regaining confidence with education and friendships.

**Case description:**

A 13 year old girl (HA) attended clinic in a wheelchair with her Mother. She is one of five children and she also has a twin sister who is well.

HA developed pains in her legs when she was seven years old. Initially the pain was in her legs at the end of the day and after activity. She reduced her activities and stopped doing PE. HA developed burning pain in her palms and soles. When this pain occurred her soles and palms would also turn red. The pain lasts between 10 mins to a few hours and is eased by using cold objects or water.

HA was seen by GP, paediatrician and Physiotherapy locally. She was found to be hypermobile and was given physio. She was reporting increasing pain and she was scared to go out or attend school and so she was given crutches and a wheelchair. She was referred to psychology.

On assessment in clinic HA was hypermobile in all joints with no evidence of joint swelling or stiffness. Muscle strength testing demonstrated MMT8 of 58/80 and MMT3 of 13/30. Gait was abnormal. She attended 50% school and only had her twin sister’s friends. Investigations were sent off for Fabry’s disease and Erythromelalgia, no results as of yet.

Treatment has been started and she has been given Lidocaine patches, pain management strategies, TENS machine and intensive physiotherapy.

It has been explained to the family that there is limited medical management for either of these conditions and the main focus of treatment is following a biopsychosocial model and regaining full muscle strength and independent mobility without the use of wheelchair, to attend fulltime school and participate in all activities. She was provided with further pain management education within a group setting and induvial sessions with the psychologist.

**Discussion:**

On assessment it is important to keep an open mind when listening to the symptoms presented as it would be easy to relate the loss of function and school attendance to a functional disorder alone. It is important to remember that there can be a combination of a pathology alongside a functional presentation with less effective self management strategies.

Both Erythromelalgia and Fabry’s have a genetic test, but this often takes many months to get a result. Both conditions are managed with a biopsychosocial approach with careful use of medication to avoid use of opiods. The goal of all treatments is to ensure that the young person remains fully mobile and independent despite the pain. This is especially difficult with these rare conditions as increased stress of physical stress can increase the severity of the pain. With a carefully paced progressive resisted exercise programme the optimum strength and stamina of muscles and of the body can be carefully increased and therefore the resilience for the young person to activity also increases.

Discussion around the use of medication in management of these pain conditions is interesting as there is a difference between the approach of a pain team vs a rheumatology team.

Discussion around specific progressive exercises are important in the physiotherapy approach to managing these conditions before progressing with functional and sporting activities and exercises.

Exploring how local teams can be supported to manage the symptoms of pain without the provision of walking aides / wheelchairs which are a short term solution and result in fear avoidance behaviours and social isolation as well and loss of strength and physical function

**Key learning points:**

• When pain presentations are more unusual such as affecting palms and soles it is important to consider these very rare pain conditions whilst balancing treatments and advice that maximise the patients function and potential.

• The young person also needs the support of a team who understands the pain but is also confident to support the approach of maintaining full function. Though it is tempting to offer the help of crutches and a wheelchair this increases the problems and loss of function. It also reduces strength, stamina and resilience resulting in more severe pain attacks.

• Physiotherapy needs to focus on regaining full strength and stamina of the individual muscle groups and ensuring that there is optimum muscle control throughout the hypermobile range before the progression onto functional activities.

• Supporting the young person to develop effective pain management strategies are important and they need to realise that these need to be practiced and explored to find an effective repertoire of techniques. These can be explored by many professionals involved and resources provided. Though some patients find breathing exercises very effective there are many other techniques and these can be explored.

• A multidisciplinary approach is important and this requires each professional to have the same message. This is often difficult in the community to achieve and then there is the risk that one message is about managing pain and the other is about resting and avoiding activity and pain and it is very confusing for the young person and the family.

